# Sublingual Cyclobenzaprine Approval: Expanding Options for Fibromyalgia Management

**DOI:** 10.1002/hsr2.72850

**Published:** 2026-07-20

**Authors:** Muhammad Ali Abid, Muhammad Hafi Abid, Abdul Haseeb Hasan, Muhammad Daniyal Afzal

**Affiliations:** ^1^ Department of Medicine King Edward Medical University Lahore Pakistan; ^2^ Department of Medicine Faisalabad Medical University Faisalabad Pakistan; ^3^ Department of Medicine Kyrgyz State Medical Academy Bishkek Kyrgyzstan

**Keywords:** cyclobenzaprine, FDA approval, fibromyalgia, sublingual cyclobenzaprine

## Abstract

**Background and Aims:**

Fibromyalgia (FM) is a chronic disorder characterized by widespread pain, fatigue, sleep disturbances, and cognitive issues. It is a multifactorial condition with no single definitive cause, making diagnosis challenging. Current treatments provide only limited relief, leading to significant unmet medical needs. Recently, the US FDA approved sublingual cyclobenzaprine (sCBP) for the treatment of FM, marking the first newly FDA‐approved medication for FM in over 15 years. This article aims to explore the current understanding of FM and the pharmacological properties, clinical trials, and potential benefits of sCBP in FM management, as well as the risks associated with its use and future research directions.

**Methods:**

We searched for relevant pieces of literature addressing FM management and the development of sublingual cyclobenzaprine. Articles were selected based on their relevance to the study objectives, and the perspective presented in this manuscript was developed through the synthesis of the available evidence and findings reported in these sources.

**Results:**

Clinical trials demonstrated that sCBP significantly reduced pain intensity in FM patients in two of the three studies (RELIEF and RESILIENT), with statistically significant mean differences in 11‐point numeric rating scale (NRS) scores compared to placebo. sCBP showed a higher peak CBP level with lower norCBP levels, supporting its enhanced pharmacokinetic profile over oral CBP. Common side effects of sCBP included oral discomfort, dry mouth, and abnormal taste, while more serious risks such as serotonin syndrome and arrhythmia were identified. Safety concerns necessitate caution when prescribing sCBP to patients with specific comorbidities or those on other serotonergic medications.

**Conclusion:**

The approval of sCBP marks a significant advancement in the treatment of FM, addressing the limitations of current pharmacological options. As the prevalence of FM continues to rise, sCBP offers a promising therapeutic alternative, providing patients with a more effective and rapid treatment option for pain management.

## 1 Introduction

Fibromyalgia (FM) is a chronic condition marked by widespread pain, sleep disturbances, fatigue, cognitive issues, depression, and anxiety [1, 2]. FM has a reported prevalence ranging from 0.2% to 4.7% worldwide, reflecting heterogeneity in geographic populations, case ascertainment methods, and evolving diagnostic criteria applied across epidemiological studies [3], with higher prevalence in women compared to men in the ratio of 3:1 [1]. The lack of specific biomarkers or laboratory tests makes FM a diagnosis of exclusion, which leads to variability in the diagnosis of FM among clinicians [1]. Overreliance on self‐reported symptoms may contribute to overdiagnosis, while stringent diagnostic criteria lead to underdiagnosis [1]. The origins of FM remain partially unclear, lacking a single defining cause [1]. FM is believed to stem from a multifaceted combination of neuroendocrine and immunological disruptions, genetic susceptibility, and environmental triggers [1]. For now, no consensus exists on its exact cause, but the pain in FM is believed to stem from faulty signaling pathways in the peripheral and central nervous system, leading to sensitization [1]. Studies have shown that the threshold needed to elicit a pain response in FM is about 50% lower than healthy controls [4]. In fibromyalgia, the central nervous system becomes hypersensitized, causing neurons in the spinal cord and brain to overreact to pain signals, which results in heightened pain responses and discomfort from normally harmless stimuli [5, 6, 7]. This is further worsened by a breakdown in the brain's natural pain‐suppressing mechanisms, imbalances in key neurotransmitters (e.g., serotonin and norepinephrine), and immune cell activation that releases inflammatory substances, collectively keeping nociceptive pathways in a persistently overactive state [5, 6].

Commonly prescribed medications for the treatment of FM include duloxetine and milnacipran (serotonin‐norepinephrine reuptake inhibitors), amitriptyline (tricyclic antidepressant), pregabalin and gabapentin (antiepileptics), and tramadol (opioid analgesic) [8]. Nonsteroidal anti‐inflammatory drugs are commonly prescribed for FM treatment, but their use has not been associated with any significant improvement in FM symptoms according to a 2017 Cochrane review [8, 9, 10]. Additionally, nonpharmacological strategies, including patient education, psychological therapies, structured physical activity, and nutritional adjustments, may help reduce pain while enhancing overall quality of life in FM as well [8].

Recent large‐scale patient survey data continue to highlight substantial dissatisfaction with FM care despite ongoing treatment. In a nationwide survey including 1626 patients with FM, overall satisfaction with pain‐related healthcare was low, with only 21.5% of patients reporting satisfaction, while nearly half (46.9%) expressed dissatisfaction with the management of their pain [11]. Many patients reported persistent unmet needs, including insufficient information about the cause of their pain, inadequate guidance on symptom management, and limited regular follow‐up [11]. Furthermore, more than half of patients reported uncertainty regarding how to improve their condition, reflecting ongoing gaps in patient education and treatment effectiveness [11]. Delayed diagnosis is another significant contributor to socioeconomic burden on both the patients and the health system, with the average time from symptom onset to diagnosis being approximately 6 years [12]. Recent patient experiences have also revealed that most patients were unable to adhere to their treatment protocols due to concerns ranging from fear of addiction, difficulty tolerating side effects, and worries about the risk of suicidal thoughts associated with antidepressant use [12]. Collectively, these findings underscore the persistent burden of uncontrolled symptoms and reinforce the need for additional therapeutic strategies to improve clinical outcomes in FM.

### 1.1 Approval of Sublingual Cyclobenzaprine

Recently, the US Food and Drug Administration (FDA) has approved TONMYA, sublingual cyclobenzaprine (sCBP) for FM, which is the first new FDA‐approved treatment for FM in over 15 years [13]. Previously, the medical treatment of FM as approved by the FDA included duloxetine, pregabalin, and milnacipran [2]. The recommended dosage of sCBP is 2.8 mg once daily (1 sublingual tablet) at bedtime from Day 1–14, which is then increased to 5.6 mg once daily (2 sublingual tablets) at bedtime from Day 15 and onwards [13, 14]. The maximum recommended dosage in a day is 5.6 mg [13, 14]. For geriatric patients or those suffering from mild hepatic dysfunction, the maximum daily dose should be 2.8 mg [13, 14]. The tablet should be placed under the tongue after brushing teeth and completing other oral care, and the patient must not eat or drink anything for at least 15 min after the tablet(s) have completely dissolved [13, 14].

### 1.2 Mechanism of Action and Pharmacokinetics

Cyclobenzaprine (CBP) is a strong antagonist of post‐synaptic receptors (adrenergic‐α_1_, histaminergic‐H_1_, serotonergic‐5‐HT_2A_ and muscarinic‐M_1_‐cholinergic) each of which plays a significant role in sleep regulation [13, 15]. CBP is also a weak inhibitor of norepinephrine and serotonin transporters [15]. Despite this, oral CBP only provides transient relief for FM pain, this is due to the hepatic first pass metabolism that converts CBP to norcyclobenzaprine (norCBP), an active secondary amine tricyclic metabolite [15]. As compared to CBP, norCBP is a weaker antagonist at α_1_, 5‐HT_2A_, H_1_, and M_1_ receptors while being a strong inhibitor at norepinephrine transporter, potentially leading to unwanted stimulatory effects [15]. The sCBP tablet was developed to bypass the hepatic first pass metabolism and ensure the rapid and effective transmucosal absorption of CBP while simultaneously minimizing the buildup of norCBP [2, 15]. Incorporating a basic excipient enhances CBP's transmucosal absorption by elevating the local concentration of CBP free base [15]. Additionally, an eutectic mixture of mannitol and CBP HCl was found to shield CBP HCl from the basifying agent, ensuring a stable tablet formation [15]. Clinical pharmacokinetic studies have revealed noteworthy benefits of sCBP compared to oral CBP for daily bedtime dosing [15]. sCBP achieved greater CBP exposure levels in the first 2 h after administration and lower norCBP levels in both single dose and multiple dose studies [15]. In a 20‐day multidose pharmacokinetic study, sCBP at steady‐state produced a higher peak CBP level relative to norCBP level [15]. Conversely, a simulated 20‐day pharmacokinetic analysis of oral CBP showed norCBP concentration to be greater than peak CBP levels [15]. This aligns with sCBP absorption bypassing hepatic first pass metabolism [2, 15].

## 2 Results From Trials

The FDA approval of sCBP is based on the data from three randomized, two‐arm, parallel‐group, double‐blind placebo‐controlled trials [13, 14]. A total of 1474 patients were included who met the 2016 American College of Rheumatology (ACR) criteria for diagnosis of FM [13]. The mean age of the patients was 49 years (ranging from 18 to 65 years), and the mean duration of FM in patients was 9 years (ranging from 0 to 49 years) [13]. The participants were randomly assigned to receive either a placebo every night for 14 weeks (*n* = 739), or 2.8 mg nightly sCBP for the first 2 weeks and then 5.6 mg starting on the evening of the 15th day through Week 14 (*n* = 735) [13]. The primary endpoint was set as the change in baseline to Week 14 in the weekly average of daily 24‐h recall pain intensity scores, using 11‐point (0–10) numeric rating scale (NRS). Trial 1 (RELIEF; ClinicalTrials.gov Identifier: NCT04172831) [16] and Trial 3 (RESILIENT; ClinicalTrials.gov Identifier: NCT05273749) [17] showed statistically significant reduction in pain intensity scores of sCBP as compared to the placebo (NRS scores compared with placebo least squares mean difference: Trial 1: −0.4 [95% CI, −0.7, −0.1]; *p* = 0.010; Trial 3: −0.7 [95% CI, −1.0, −0.3]; *p* < 0.001) [13, 14]. Clinical interpretation of these findings requires consideration of the minimal clinically important difference (MCID) for pain reduction in FM. Prior studies suggest that an improvement of approximately 2 points on the 11‐point numeric rating scale, or a 30% reduction in pain intensity, is generally perceived by patients as clinically meaningful [18]. The mean differences observed in the RELIEF and RESILIENT trials (−0.4 and −0.7 points, respectively) therefore represent modest average treatment effects that may fall below conventional MCID thresholds at the population level. However, group mean differences may underestimate individual patient benefit, as a subset of patients may achieve clinically meaningful responses despite smaller overall averages. From a clinical decision‐making perspective, these findings suggest that sCBP should be viewed as an adjunctive or individualized treatment option rather than a universally transformative therapy, with potential value particularly in patients with sleep disturbance or intolerance to existing pharmacologic treatments.

In Trial 2 (RALLY; ClinicalTrials.gov Identifier: NCT04508621) [19], no statistically significant difference was observed between sCBP and placebo [13]. Several factors may explain this negative outcome. FM trials are particularly susceptible to high placebo response rates, which can reduce the ability to detect treatment effects despite pharmacologic activity [20, 21]. Variability in baseline symptom severity, differences in patient‐reported outcome sensitivity, and heterogeneity in FM phenotypes may also have contributed to diminished separation between treatment and placebo groups. The presence of one negative trial alongside two positive studies introduces some uncertainty regarding the consistency of effect and suggests that treatment response may be population‐dependent. Consequently, while the overall evidence supports efficacy, the mixed trial results highlight the need for further studies to better identify patient subgroups most likely to achieve clinically meaningful benefit. The summary of the three trials has been given in Table 1.

**Table 1:** Summary of pivotal Phase 3 trials evaluating sublingual cyclobenzaprine in fibromyalgia.

Trial name	ClinicalTrials.gov identifier	Study design	Sample size (*n*)	Treatment regimen	Primary endpoint	Primary outcome result	Key adverse events reported
RELIEF [16]	NCT04172831	Randomized, double‐blind, placebo‐controlled, parallel‐group Phase 3 trial	503 (TG = 248, CG = 255)	sCBP 2.8 mg nightly for 2 weeks, then 5.6 mg nightly for 12 weeks vs. placebo	Change from baseline to week 14 in weekly average daily pain intensity (11‐point NRS)	Statistically significant reduction vs. placebo (LS mean difference −0.4; 95% CI −0.7 to −0.1; *p* = 0.010)	Oral hypoesthesia, oral paresthesia, dysgeusia
RALLY [19]	NCT04508621	Randomized, double‐blind, placebo‐controlled, parallel‐group Phase 3 trial	514 (TG = 256, CG = 258)	Same dosing schedule as RELIEF	Same primary endpoint as RELIEF	No statistically significant difference (LS mean difference −0.2; 95% CI −0.6 to 0.1)	Oral hypoesthesia, oral discomfort, abnormal taste, tongue discomfort
RESILIENT [17]	NCT05273749	Randomized, double‐blind, placebo‐controlled, parallel‐group Phase 3 trial	457 (TG = 231, CG = 226)	Same dosing schedule as RELIEF	Same primary endpoint as RELIEF	Statistically significant reduction vs. placebo (LS mean difference −0.7; 95% CI −1.0 to −0.3; *p* < 0.001)	Oral hypoesthesia, oral discomfort, abnormal taste, tongue discomfort

Abbreviations: CG, control group; CI, confidence interval; LS, least squares; NRS, numeric rating scale; sCBP, sublingual cyclobenzaprine; TG, treatment group.

Interpretation of treatment effects in FM trials must also consider the consistently high placebo response observed in this condition [20, 21]. Placebo‐associated improvements in pain and functional outcomes are well documented in FM, and are thought to reflect fluctuating symptom severity, patient expectations, intensive trial follow‐up, and the subjective nature of patient‐reported pain measures [21]. Reported placebo response rates in FM can reduce detectable between‐group differences even when active therapies provide a biological benefit. Consequently, modest mean differences between treatment and placebo groups should be interpreted cautiously and alongside responder analyses and patient‐centered outcomes rather than as sole indicators of clinical value.

### 2.1 Safety Considerations

Although sCBP has great potential in the management of FM, its use poses several risks that should be considered when counseling FM patients for its use. Cyclobenzaprine is contraindicated in patients with known hypersensitivity to the drug or any of its excipients, which may present as anaphylaxis, urticaria, angioedema, or pruritus, and the drug should be promptly discontinued upon any suspected hypersensitivity reaction [13]. Concurrent use with monoamine oxidase inhibitors (MAOI), or within 14 days of their discontinuation, is strictly contraindicated due to the risk of hyperpyretic crisis, seizures, and potentially fatal outcomes [13]. Additionally, cyclobenzaprine should not be administered during the acute recovery phase following myocardial infarction, nor in patients with pre‐existing arrhythmias, cardiac conduction disturbances, heart block, or congestive heart failure [13]. Hyperthyroidism also represents a contraindication to its use [13].

The most common side effects reported with CBP use are dry mucous membranes (xerostomia), dizziness, confusion, and somnolence [22]. The most common side effects of sCBP reported in Trial 1, Trial 2, and Trial 3 were abnormal product taste, oral hypoesthesia, oral paresthesia, tongue discomfort, dry mouth, and aphthous ulcer [13]. The more serious side effect that physicians prescribing sCBP need to be on the lookout for is serotonin syndrome [13, 22]. It can develop if the patient is taking other serotonergic medications like selective serotonin reuptake inhibitors (SSRIs), serotonin‐norepinephrine reuptake inhibitors (SNRIs), and tricyclic antidepressants (TCAs) alongside sCBP. Due to similarities in structure to tricyclic antidepressants, sCBP may cause tachycardia or arrhythmia [13]. Its atropine‐like side effects require caution when using sCBP in patients with angle closure glaucoma, urinary retention, or those on anticholinergic drugs [13]. Animal reproductive toxicity findings described in the prescribing information report neural tube defects in a rabbit embryofetal development study [13]. Consequently, use during the peri‐conception period and first trimester is discouraged, and pregnancy testing and effective contraception are recommended during treatment and for 2 weeks after discontinuation [13]. sCBP monotherapy may cause CNS depression, and concurrent use with other alcohol and CNS depressants may further increase the risk [13]. Overall, the trials showed a significant decrease in pain with the use of sCBP as compared to placebo in the patients.

### 2.2 Comparison With Current FDA‐Approved Fibromyalgia Treatments

The clinical relevance of sCBP is best interpreted alongside the three established FDA‐approved pharmacotherapies for FM (duloxetine, pregabalin, and milnacipran) [8]. Across FM trials, average improvements in pain are often modest at the group level, and comparative reviews suggest broadly similar short‐term efficacy for these agents, with differential effects on symptom domains (e.g., mood, sleep, fatigue) rather than uniformly large analgesic effects. In a Bayesian network meta‐analysis, duloxetine was found to be more efficacious than pregabalin and milnacipran [23]. Direct head‐to‐head comparisons between sCBP and the previously approved pharmacotherapies remain limited, and additional comparative studies are needed to better define their relative efficacy and clinical value.

Safety and tolerability profiles differ meaningfully and may drive real‐world selection more than efficacy differences. Duloxetine is associated with adverse effects like nausea, constipation, dizziness, insomnia, and decreased appetite [24]. It also carries important safety considerations, including a boxed warning for suicidality in younger populations and risks of serotonin syndrome and hepatotoxicity [24]. Commonly observed side effects associated with milnacipran are nausea, headache, and constipation [25]. Pregabalin is often limited by dizziness and somnolence and may cause weight gain and peripheral edema, which can be problematic in patients with cardiac comorbidity [26]. In this context, sCBP offers a differentiated mechanism and delivery strategy that primarily targets sleep‐related and neuromodulatory pathways while avoiding extensive first‐pass metabolism, however, the observed mean pain differences versus placebo are modest, and adverse effects such as oral hypoesthesia/paresthesia, abnormal taste, and dry mouth may affect adherence [13].

### 2.3 Future Directions

The approval of sCBP represents a promising advancement in the treatment of FM, but several areas require further exploration to optimize its use and improve patient outcomes. Studies which explore whether the benefits observed in the short term persist and whether it helps mitigate disease progression or flare‐ups over time will be crucial. Additionally, real‐world data and patient‐reported outcomes will provide insights into the effectiveness of sCBP in clinical settings. These studies can reveal information about patient adherence, the side effect profile, and improvements in overall quality of life, which will help healthcare providers make more informed treatment decisions. Investigating combination therapies that include sCBP along with other FM treatments, such as duloxetine or pregabalin, may offer better symptom management, especially since FM patients often experience a combination of pain, fatigue, and sleep disturbances. Research into the safety and efficacy of sCBP in pediatric and adolescent populations is also necessary, as FM can affect younger individuals [27], and determining age‐appropriate dosing and side effects is essential. Moreover, cost‐effectiveness studies are crucial to evaluate the affordability of sCBP, ensuring it is accessible in low‐resource settings. Finally, investigating whether ethnic and gender differences impact the effectiveness of sCBP will be important in ensuring that treatment plans can effectively cater to diverse patient populations. Overall, while sCBP holds significant promise, ongoing research and continuous evaluation of its long‐term effects, safety, and efficacy are vital to enhancing its role in managing FM.

## 3 Conclusion

In conclusion, the approval of sCBP marks a significant step forward in FM treatment, offering an additional therapeutic alternative to current therapies [28]. By bypassing hepatic first‐pass metabolism, sCBP provides faster and more consistent pain relief compared to oral cyclobenzaprine. While clinical trials have shown its potential in reducing pain intensity, variability across studies calls for further research into its long‐term efficacy and safety. Despite its promise, sCBP has associated risks, such as serotonin syndrome and cardiovascular effects, requiring careful monitoring. Future research should focus on real‐world data, combination therapies, and use in diverse populations. With ongoing evaluation, sCBP could become a key treatment in FM management, improving patient outcomes and quality of life.

## Author Contributions

**Muhammad Ali Abid:** supervision, conceptualization, project administration, writing – original draft, writing – review and editing, formal analysis, investigation, resources. **Muhammad Hafi Abid:** conceptualization, writing – original draft, writing – review and editing. **Abdul Haseeb Hasan:** Writing – original draft, writing – review and editing. **Muhammad Daniyal Afzal:** validation, writing – review and editing.

## Funding

The authors have nothing to report.

## Ethics Statement

The authors have nothing to report.

## Conflicts of Interest

The authors declare no conflicts of interest.

## Transparency Statement

The lead author, Muhammad Ali Abid, affirms that this manuscript is an honest, accurate, and transparent account of the study being reported, that no important aspects of the study have been omitted, and that any discrepancies from the study as planned (and, if relevant, registered) have been explained.

## Data Availability

Data sharing not applicable to this article as no datasets were generated or analyzed during the current study.
